# Accuracy of Glucagon Testing Across Transition in Young Adults With Childhood-Onset GH Deficiency

**DOI:** 10.1210/clinem/dgae408

**Published:** 2024-06-24

**Authors:** Daniela Fava, Davide Guglielmi, Carlotta Pepino, Alessia Angelelli, Emilio Casalini, Carolina Varotto, Marta Panciroli, Caterina Tedesco, Tiziana Camia, Alessandro Naim, Anna Elsa Maria Allegri, Giuseppa Patti, Flavia Napoli, Roberto Gastaldi, Stefano Parodi, Mariacarolina Salerno, Mohamad Maghnie, Natascia Di Iorgi

**Affiliations:** Department of Neuroscience, Rehabilitation, Ophthalmology, Genetics, Maternal and Child Health, University of Genoa, 16132 Genoa, Italy; Pediatric Endocrinology Unit, Department of Pediatrics, IRCCS Istituto Giannina Gaslini, 16147 Genoa, Italy; Pediatric Endocrinology Unit, Department of Pediatrics, IRCCS Istituto Giannina Gaslini, 16147 Genoa, Italy; Department of Neuroscience, Rehabilitation, Ophthalmology, Genetics, Maternal and Child Health, University of Genoa, 16132 Genoa, Italy; Department of Neuroscience, Rehabilitation, Ophthalmology, Genetics, Maternal and Child Health, University of Genoa, 16132 Genoa, Italy; Pediatric Endocrinology Unit, Department of Pediatrics, IRCCS Istituto Giannina Gaslini, 16147 Genoa, Italy; Department of Neuroscience, Rehabilitation, Ophthalmology, Genetics, Maternal and Child Health, University of Genoa, 16132 Genoa, Italy; Pediatric Endocrinology Unit, Department of Pediatrics, IRCCS Istituto Giannina Gaslini, 16147 Genoa, Italy; Department of Neuroscience, Rehabilitation, Ophthalmology, Genetics, Maternal and Child Health, University of Genoa, 16132 Genoa, Italy; Department of Neuroscience, Rehabilitation, Ophthalmology, Genetics, Maternal and Child Health, University of Genoa, 16132 Genoa, Italy; Pediatric Endocrinology Unit, Department of Pediatrics, IRCCS Istituto Giannina Gaslini, 16147 Genoa, Italy; Department of Neuroscience, Rehabilitation, Ophthalmology, Genetics, Maternal and Child Health, University of Genoa, 16132 Genoa, Italy; Department of Neuroscience, Rehabilitation, Ophthalmology, Genetics, Maternal and Child Health, University of Genoa, 16132 Genoa, Italy; Pediatric Endocrinology Unit, Department of Pediatrics, IRCCS Istituto Giannina Gaslini, 16147 Genoa, Italy; Department of Neuroscience, Rehabilitation, Ophthalmology, Genetics, Maternal and Child Health, University of Genoa, 16132 Genoa, Italy; Pediatric Endocrinology Unit, Department of Pediatrics, IRCCS Istituto Giannina Gaslini, 16147 Genoa, Italy; Pediatric Endocrinology Unit, Department of Pediatrics, IRCCS Istituto Giannina Gaslini, 16147 Genoa, Italy; Pediatric Endocrinology Unit, Department of Pediatrics, IRCCS Istituto Giannina Gaslini, 16147 Genoa, Italy; Epidemiology and Biostatistics Unit, Scientific Directorate, IRCCS Istituto Giannina Gaslini, 16147 Genoa, Italy; Department of Translational Medical Sciences, University Federico II, 80138 Naples, Italy; Department of Neuroscience, Rehabilitation, Ophthalmology, Genetics, Maternal and Child Health, University of Genoa, 16132 Genoa, Italy; Pediatric Endocrinology Unit, Department of Pediatrics, IRCCS Istituto Giannina Gaslini, 16147 Genoa, Italy; Department of Neuroscience, Rehabilitation, Ophthalmology, Genetics, Maternal and Child Health, University of Genoa, 16132 Genoa, Italy; Pediatric Endocrinology Unit, Department of Pediatrics, IRCCS Istituto Giannina Gaslini, 16147 Genoa, Italy

**Keywords:** GH deficiency, transition, glucagon, ITT, young adults, brain tumors, pituitary stalk interruption syndrome, congenital hypopituitarism, posterior pituitary ectopia

## Abstract

**Context:**

The 2019 American Association of Clinical Endocrinologists guidelines suggested peak GH-cutoffs to glucagon test (GST) of ≤3 and ≤1 µg/L in the diagnosis of permanent GH deficiency (GHD) during the transition phase.

**Objective:**

The aim of the study was to evaluate the accuracy of GST compared to insulin tolerance test (ITT) in the definition of GHD at adult height achievement.

**Patients and methods:**

Ninety-seven subjects with childhood-onset GHD (median age, 17.39 years) underwent ITT, GST, and IGF-1 testing; 44 subjects were idiopathic (isolated GHD), 35 moderate organic GHD (0-2 hormone deficiencies) and 18 severe organic GHD (≥3 hormone deficiencies).

**Results:**

Bland and Altman analysis showed a high consistency of GH peak measures after ITT and GST. Receiver operating characteristic analysis identified 7.3 μg/L as the optimal GH peak cutoff to GST [95% confidence interval (CI) 4.15-8.91; sensitivity 95.7%, specificity 88.2%, positive predictive value (PPV) 88.0%, negative predictive value (NPV) 95.7%] able to correctly classify 91.8% of the entire cohort while 5.8 μg/L was the best GH peak cutoff able to correctly classify 91.4% of moderate organic GHD patients (95% CI 3.16-7.39; sensitivity 96.0%, specificity 80.0%, PPV 92.3%, NPV 88.9%). Patients with ≥3 hormone deficiencies showed a GH peak <5 μg/L at ITT and <5.8 μg/L at GST but 1. The optimal cutoff for IGF-1 was −1.4 SD score (95% CI −1.94 to 0.77; sensitivity 75%, specificity 94%, PPV 91.7%, NPV 81.0%) that correctly classified 85.1% of the study population.

**Conclusion:**

A GH peak to GST <5.8 μg/L represents an accurate diagnostic cutoff for young adults with childhood-onset GHD and high pretest probability of permanent GHD.

Several studies have reported that patients with childhood-onset GH deficiency (CO-GHD) can “normalize” GH secretion when reevaluated at the end of growth, while others show permanent or evolving pituitary defects, especially in subjects with congenital structural hypothalamic-pituitary anomalies or acquired brain lesions; this evidence suggests the need for retesting before transitioning to adult care (1-10). Specifically, children with isolated GHD and normal pituitary anatomy or small pituitary gland on brain magnetic resonance imaging (MRI) are more likely to have reversal of GH dysfunction, while those with neuroimaging abnormalities are at greater risk for lifelong need for replacement treatment of pituitary defects (11-15).

Although it is recognized that the diagnosis of permanent GHD is crucial, recommendations to confirm or exclude GHD after reaching adult stature are still debated as to what limit for a normal response should be considered in this age group after various stimulation tests and under different conditions, including cancer survivors (16-23). The reported GH cutoff value (≤6 µg/L) after the insulin tolerance test (ITT) for GHD in young adults in 2005 (16) was subsequently recommended for the diagnosis of GHD during the transition period from the 2007 international consensus statement (18); this indication was later confirmed by other studies (12, 13), and the value of 6 µg/L was accepted by the Italian Medicines Agency as a GH cutoff for the diagnosis of GHD in transition age.

Despite the recognition of ITT as a gold standard test, it is contraindicated in patients with a history of seizures, epilepsy, or cerebral or cardiovascular disease (17). Alternatively, different GH cutoff values have been proposed for the combined GH-releasing hormone plus arginine test (GHRH-A), and the results have shown that the test may be unreliable in diagnosing children and young adults with CO-GHD of different etiologies or weight (19-29). Consequently, a missed or uncertain diagnosis could have consequences on health and quality of life since young adults with CO-GHD who have discontinued therapy with recombinant human growth hormone (rhGH) have lower bone mineral density, abnormal bone micro-architecture, and increased fracture risk compared to those without GHD or with adult-onset GHD (30-32). Moreover, in untreated young adults with GHD, discontinuation of rhGH treatment for 2 years after transition induced significant and unfavorable modifications in waist circumference, body composition, and serum fibrinogen (12), while long-term therapy improved lipid metabolism, body composition, bone health, and quality of life (33).

According to the 2019 American Association of Clinical Endocrinologists (AACE) guidelines, continuation of rhGH treatment is recommended without provocative GH testing if there is biochemical evidence of multiple pituitary hormone deficiencies (MPHDs) (≥3 pituitary hormone deficiencies) and low serum IGF-1 levels [<−2.0 SD score (SDS)] (34). Conversely, confirmation of the diagnosis requires a GH stimulation test, including ITT or GST, for patients with isolated GHD and IGF-1 level < 0 SDS; in these patients, if IGF-1 level is ≥0 SDS, testing is not required since a permanent defect is unlikely (34). Noticeably, the diagnostic cutoff values of GHD after GST suggested by the AACE guidelines based on the high or low pretest probability and on the body mass index (BMI) (≤3 and ≤1 µg/L) (34) have not yet been confirmed in clinical practice during the transition phase.

The aim of the study was to evaluate the diagnostic value of the GST compared to ITT in the diagnosis of GHD in young adults with CO-GHD treated with rhGH until adult height achievement.

## Methods

### Patients’ Characteristics

Ninety-seven patients (62 males, 35 females) with CO-GHD, including 35 with brain tumors, underwent an ITT (intravenous bolus of 0.1 IU/kg human regular insulin) and a GST (1 mg intramuscular) in 2 different days in a single tertiary level academic center (Pediatric Endocrine Unit, IRCCS Istituto Giannina Gaslini, University of Genova; Genova, Italy) from September 2013 to June 2023. All patients were retested after at least 1 month following discontinuation of rhGH therapy after reaching adult height (growth velocity <2 cm/year) and pubertal maturity (Tanner stage 4-5).

**Figure 1.**
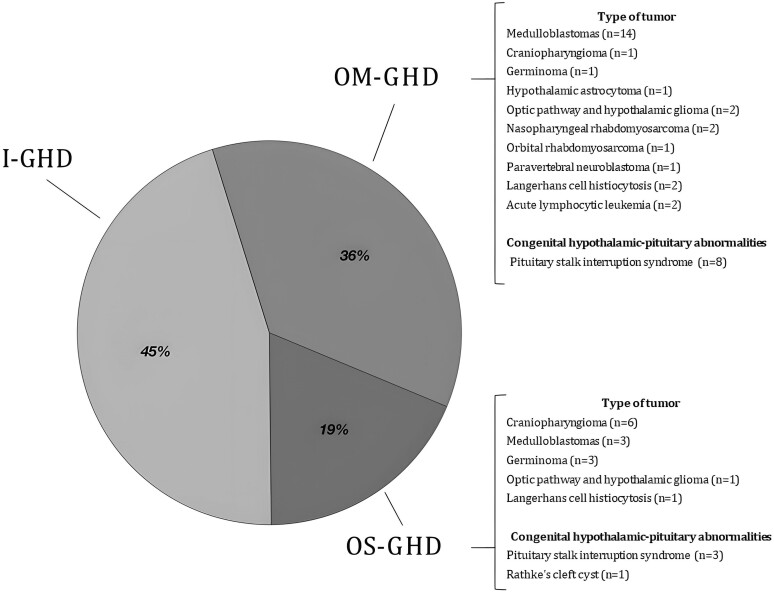
Etiological diagnosis of 97 patients with childhood-onset GHD at the time of the study subdivided in 3 groups: I-GHD, OM-GHD, OS-GHD. Abbreviations: GHD, GH deficiency; I-GHD, idiopathic growth hormone deficiency; OM-GHD, organic moderate GHD; OS-GHD, organic severe GHD.

Patients were classified into 3 groups based on MRI findings at GHD diagnosis and the number of pituitary hormone deficiencies according to the 2019 AACE Guidelines (34) (Fig. 1). In the first group, n = 44 patients (45.4%) with normal MRI findings, no history of cancer, and isolated GHD were included and considered “idiopathic” (I-GHD); these patients were considered likely to have a low pretest probability for permanent GHD. The second group comprised 35 subjects (36%): n = 8 patients with congenital anomalies (pituitary stalk interruption syndrome and other cerebral midline defects) and n = 27 with organic hypothalamic-pituitary disease [acquired following brain surgery, craniospinal (CSI), total body irradiation (TBI), or infiltrative disease of the hypothalamus and pituitary stalk]. They presented 1 or 2 pituitary defects and were defined as “organic moderate” GHD (OM-GHD); these patients were considered likely to have a high pretest probability for permanent GHD. The third group included 18 subjects with 3 or more pituitary hormone deficiencies (MPHD) (18.6%): n = 4 patients with congenital or acquired anomalies involving the hypothalamic-pituitary region and n = 14 with a central nervous system tumor: these patients were classified into the “organic severe” GHD group (OS-GHD) and retested for comparison despite they would not require GH reevaluation based on the current recommendations (34).

Twenty of the OM-GHD patients had an isolated GHD and 15 had 2 pituitary defects, while among OS-GHD patients, 4 had panhypopituitarism, 5 had 4 pituitary defects including GHD, and 9 patients had 3 pituitary defects. At the time of retesting, all patients with hormone deficiencies were receiving replacement therapy with hydrocortisone (7-9 mg/m^2^/day) and/or L-thyroxine and/or desmopressin acetate (range 30-120 mcg 3 times a day orally), as needed. Male patients with hypogonadism (n = 4) were receiving testosterone intramuscularly (200-250 mg/every 3 weeks), while females with hypogonadism (n = 8) were on hormone replacement therapy (HRT) consisting of oral or transdermal estrogens for 21 days per month (ethinylestradiol up to 30 mcg/day in 2 subjects or 17β-estradiol 50 mcg/day in 6 patients, respectively) and oral medroxyprogesterone acetate (10 mg/day) for 11 days a month.

The diagnosis of GHD in childhood and adolescence was established in the presence of clinical criteria and a low GH response to 2 different provocative tests after an overnight fast; we considered a peak GH concentration after ITT, arginine, GST below 10 µg/L as insufficient until 2014 and less than 8 µg/L thereafter, according to national recommendations.

The study was approved by the Ethics Committee Genoa, Italy (Protocol Number 13777/21), and written informed consent was obtained from parents or caregivers of patients after a full explanation of the study according to the Declaration of Helsinki.

### Data Collection

We retrospectively collected clinical and treatment data (date of birth, cancer diagnosis, oncological treatments, date of GHD diagnosis and of additional pituitary defects, HRT information, date of GH retesting), anthropometric parameters based on Tanner charts (35-37) (height, height SDS, pubertal Tanner stage) and biochemical data including baseline IGF-1 and insulin. BMI SDS was calculated according to Cole et al (38). All these data were recorded at the time of GHD diagnosis (Table 1) and of retesting (Table 2). At GHD diagnosis, all subjects included in the study underwent brain MRI examination and a high-resolution sellar MRI that provided detailed information of the suprasellar compartment and of the pituitary stalk. Bone age (BA) at diagnosis was assessed according to Greulich and Pyle (38) and reevaluated by the same expert endocrinologist (D.F.) at the time of the study. The delta BA for chronological age (ΔBA/CA) was calculated. Parental height measurements, when available, were used to calculate target height (TH) SDS (mean of parental height/2 ± 6.5 cm) and delta TH-H SDS; IGF-1 was expressed in SDS based on normative data (39, 40).

**Table 1.** Characteristics of 97 patients with childhood-onset GHD at the time of diagnosis

	Study population	I-GHD	OM-GHD	OS-GHD	*P* value
Clinical characteristics					
No. of patients (males/females)	97 (62/35)	44 (33/11)	35 (20/15)	18 (9/9)	.10
Age (years)	11.37 (8.55 to 13.02)	12.02 (9.76 to 13.80)	10.67 (8.23 to 12.53)	9.86 (7.06 to 12.12)	.0306
Height SDS	−2.04 (−2.60 to −1.30)	−2.35 (−2.80 to −2.00)	−1.61 (−2.30 to −0.60)	−0.80 (−1.58 to 0.10)	.0001
BMI SDS	0.50 (−0.70 to 1.60)	−2.00 (−1.00 to 1.30)	0.80 (−0.52 to 1.86)	1.80 (0.34 to 2.30)	.0227
Delta H-TH SDS	−1.40 (−2.30 to −0.70)	−1.80 (−2.34 to −1.21)	−0.93 (−2.70 to −0.30)	−0.90 (−1.48 to −0.20)	.0150
Delta CA-BA	−1.08 (−2.00 to −0.29)	−1.46 (−2.43 to −0.90)	−0.65 (−1.61 to −0.05)	−0.90 (−1.75 to 0.80	.0608
Peak GH on provocative test					
Arginine (μg/L) (n = 80)	4.17 (2.18 to 6.17)	5.89 (3.31 to 6.94)	3.28 (1.79 to 4.61)	2.93 (0.08 to 4.66)	.0014
ITT (μg/L) (n = 73)	3.49 (1.74 to 4.80)	4.33 (3.21 to 5.27)	2.80 (1.57 to 4.53)	1.19 (0.59 to 1.90)	.0001
GST (μg/L) (n = 21)	3.82 (2.11 to 5.96)	6.48 (4.02 to 7.525)	2.50 (1.65 to 3.82)	2.41 (1.08 to 6.01)	.0118
Biochemical data					
IGF-1 SDS (n = 82)	−1.94 (−2.67 to −1.00)	−1.79 (−2.5 to −0.82)	−1.96 (−2.6 to −1.09)	−3.31 (−4.30 to −1.84)	.0457
Glycated hemoglobin (%)	5.14 (4.98 to 5.35)	5.19 (5.02 to 5.38)	5.16 (4.84 to 5.33)	5.06 (4.96 to 5.30)	.5601
Blood glucose (mg/dL)	88.0 (83.0 to 93.0)	88.0 (84.0 to 94.0)	89.0 (83.0 to 94.0)	84.0 (79.0 to 86.0)	.0828
Baseline insulin (uU/mL)	7.1 (3.6 to 11.9)	5.0 (3.10 to 9.8)	9.05 (5.5 to 16.6)	9.00 (1.4 to 14.5)	.0219

Results are expressed as median (interquartile range, 25th to 75th percentiles), unless explained otherwise.

Abbreviations: BMI, body mass index; Delta CA-BA, delta chronological age-bone age; Delta H-TH SDS, delta height-target height SD score; GHD, GH deficiency; GST, glucagon stimulation test; I-GHD, idiopathic GHD; ITT, insulin tolerance test; OM-GHD, organic moderate GHD; OS-GHD, organic severe GHD; SDS, SD score.

**Table 2.** Characteristics of 97 patients with childhood-onset GHD at the time of retesting

	Study population n = 97	I-GHD n = 44	OM-GHD n = 35	OS-GHD n = 18	*P* value
Clinical characteristics					
Age (years)	17.39 (15.87 to 18.30)	17.99 (16.47 to 18.81)	16.39 (15.23 to 17.67)	17.68 (15.97 to 18.98)	.0077
Height SDS	−1.10 (−1.80 to 0.00)	−1.10 (−1.75 to −0.50)	−1.20 (−2.00 to 0.10)	−0.10 (−1.60 to 0.60)	.0751
BMI SDS	0.60 (−0.35 to 1.60)	0.10 (−0.70 to 0.93)	0.60 (0.03 to 1.83)	1.40 (0.70 to 2.30)	.0002
Peak GH on provocative test					
ITT (μg/L)	6.31 (1.73 to 15.05)	14.03 (9.78 to 21.31)	2.34 (1.24 to 6.91)	0.80 (0.25 to 1.96)	<.0001
GST (μg/L)	6.85 (1.73 to 12.41)	12.035 (10.32 to 18.77)	3.24 (1.67 to 5.94)	0.71 (0.27 to 1.73)	.0001
Biochemical data					
IGF-1 SDS	−0.76 (−2.31 to 0.14)	0.00 (−0.59 to 0.91)	−1.96 (−2.83 to −0.58)	−2.28 (−3.77 to −1.31)	.0001
Glycated hemoglobin (%)	5.01 (4.75 to 5.25)	5.01 (4.76 to 5.16)	5.07 (4.75 to 5.34)	4.98 (4.67 to 5.21)	.5503
Blood glucose (mg/dL)	89.0 (85.0 to 94.0)	91.5 (88.0 to 94.0)	88.0 (84.0 to 96.0)	82.5 (75.0 to 87.0)	.0002
Baseline insulin (uU/mL)	9.6 (7.1 to 15.6)	9.2 (7.8 to 12.3)	9.8 (6.2 to 18.3)	11.4 (6.3 to 16.7)	.7220

Results are expressed as median (interquartile range, 25th to 75th percentiles), unless explained otherwise.

Abbreviations: Delta CA-BA, delta chronological age-bone age; Delta H-TH SDS, delta height-target height SD score; GHD, GH deficiency; GST, glucagon stimulation test; I-GHD, idiopathic GHD; ITT, insulin tolerance test; OM-GHD, organic moderate GHD; OS-GHD, organic severe GHD; SDS, SD score.

### Biochemical Evaluation

ITT and GST were performed on 2 different days, between 08.00 and 09.00 Am, after an overnight fasting. GST was administered at the dose of 1 mg intramuscular. Blood samples were obtained at times 0, 30, 60, 90, 120, 150, and 180 minutes after glucagon administration through intravenous heparin-locked line for the determination of GH, blood glucose; cortisol levels were measured at 0, 120, 150, and 180 minutes.

Insulin was administered intravenously at a dose of 0.1 U/kg. Blood samples were collected at time points (0, 30, 60, 90, and 120 minutes) to evaluate GH and glucose concentrations. The glucose nadir value recorded during ITT was below 40 mg/dL (2.2 mmol/L) in all subjects. Serum GH was measured by chemiluminescent immunometric assay (Immulite 2000, GH; Diagnostic Products Corporation, Los Angeles, CA; international reference preparation 98/574). The inter- and intraassay coefficients of variation were 4.2% to 6.6% and 2.9% to 4.6%, respectively, at GH concentrations of 2.6 to 17 µg/L. All samples were analyzed together at the same time. All serum IGF-I samples were measured by chemiluminescent immunometric assay (Immulite 2000; Diagnostic Products Corporation). The intra- and interassay coefficients of variation were 3.4% and 7.1%, respectively, and the sensitivity of the method was 2.6 nmol/L. After centrifugation at 4 °C, plasma was separated and stored at 20 °C. Insulin basal level was determined with chemiluminescence, hemoglobin A1c was measured with immunoturbidimetry, and serum glucose was measured automatically with a hexokinase catalyzed-glucose oxidase method.

### Statistical Analyses

Descriptive statistics were reported as absolute frequencies and percentages for qualitative variables and as median and interquartile range (IQR) for quantitative ones. Comparison of continuous variables between different categories was performed by the Mann–Whitney U test for 2-group comparisons and the Kruskal–Wallis test in the presence of 3 or more groups. The association between continuous variables was evaluated by the Spearman's ρ correlation coefficient. Agreement between GH peak levels after ITT and after GST was evaluated by the intraclass correlation coefficient (ICC) (41). Both variables were log-transformed to reduce their skewness and stabilize their variance. An ICC value > 0.80 was considered as satisfactory. The Bland and Altman plot and Pitman's test were applied to assess the agreement between the 2 measures (42). The analysis of the time trend of blood glucose and GH concentrations after GST was carried out in each group by plotting the corresponding means and SDs. Comparison between basal values of glucose in the 3 groups was performed by the ANOVA test. Values of GH concentrations were log-transformed to normalize their distribution, whereas no transformation of glucose values was needed because they were consistent with a normal distribution.

The potential diagnostic accuracy of the GH peak value after GST and of IGF-1 SDS marker was assessed by standard nonparametric receiver operating characteristic (ROC) analysis. The area under the ROC curve (AUC) was used as an estimate of pure accuracy, and the related 95% confidence intervals calculated by the method proposed by DeLong et al (43). The result of the GH peak after ITT was used as the gold standard for ROC analysis, assuming values <6 μg/L as test positive (16, 18). Sensitivity, specificity, positive predictive value (PPV), negative predictive value (NPV), and global diagnostic accuracy (the proportion of correctly classified patients) were assessed at the optimal cutoff on the ROC curve, which corresponded to the highest value of the Youden index (44). Ninety-five percent confidence intervals of the optimal cutoff were estimated by the bootstrap method, using 5000 bootstrapped samples for each analysis. Multivariable binary logistic regression analysis for the prediction of GH glucagon peak at transition was performed (between early GH peaks at diagnosis of CO-GHD and BMI SDS severity groups). Finally, the association between GH peak to GST and BMI SDS, adjusted by patient group, was assessed by linear regression analysis. All analyses were carried out by STATA 18.0 for Windows statistical package (Stata Corporation, College Station, TX, USA).

## Results

### Characteristics of the **S**tudy **C**ohort at CO-GHD **D**iagnosis

The clinical and biochemical characteristics and GH peak concentrations at first GHD diagnosis for each group of patients are reported in Table 1. OS-GHD patients were significantly younger, taller, and heavier compared to OM-GHD and I-GHD patients; 15 patients of the entire cohort (14.4%) had obesity (BMI ≥ 2 SDS); 40% of these belonged to the OS-GHD group, 40% to the OM-GHD group, and 20% to the I-GHD group. The OS-GHD group had the highest prevalence of obesity, with 33.3% having a BMI ≥ 2 SDS. In comparison, 17.4% of the OM-GHD group and 6.8% of the I-GHD group had obesity at GHD diagnosis. The Delta H-TH was significantly wider in I-GHD, and Delta BA-CA tended to be higher in these patients. OS-GHD patients showed similar GH peak values compared to OM-GHD but lower IGF-1 levels.

### Characteristics of the **S**tudy **C**ohort at GHD **R**etesting

The median age at GHD retesting was 17.39 years (IQR 15.87; 18.30); OS-GHD were the tallest and had the highest BMI SDS (*P* = .0002), with OM-GHD patients being the youngest (Table 2). Fifteen patients of the entire cohort (15.4%) had obesity (BMI ≥ 2 SDS); in particular, 6 patients out of 18 (33.3%) had obesity in OS-GHD group; 5 had craniopharyngioma and 1 had Langerhans cell histiocytosis. Seven patients out of 35 (20%) had obesity in the OM-GHD group and 5 of them (71.4%) had been diagnosed with brain tumors or had been treated with radiotherapy. Glycated hemoglobin was within the normal range, and type 2 diabetes was excluded in all subjects.

Forty-one patients of the entire cohort (42.3%) survived from childhood cancer (CCS); 14 were in the OS-GHD and 27 in the OM-GHD group. Thirty-five had brain tumors (14 in OS-GHD and 21 in OM-GHD group). Thirty-one received radiotherapy (8 in OS-GHD, 23 in OM-GHD group), 29 patients underwent CSI, and 2 received TBI. The median time between radiotherapy stop and GHD retesting was 9.97 years (IQR 7.42;12.42). Ten of 41 CCS patients (24.4%) had obesity; they represent 76.9% of patients with obesity in the entire cohort.

**Figure 2.**
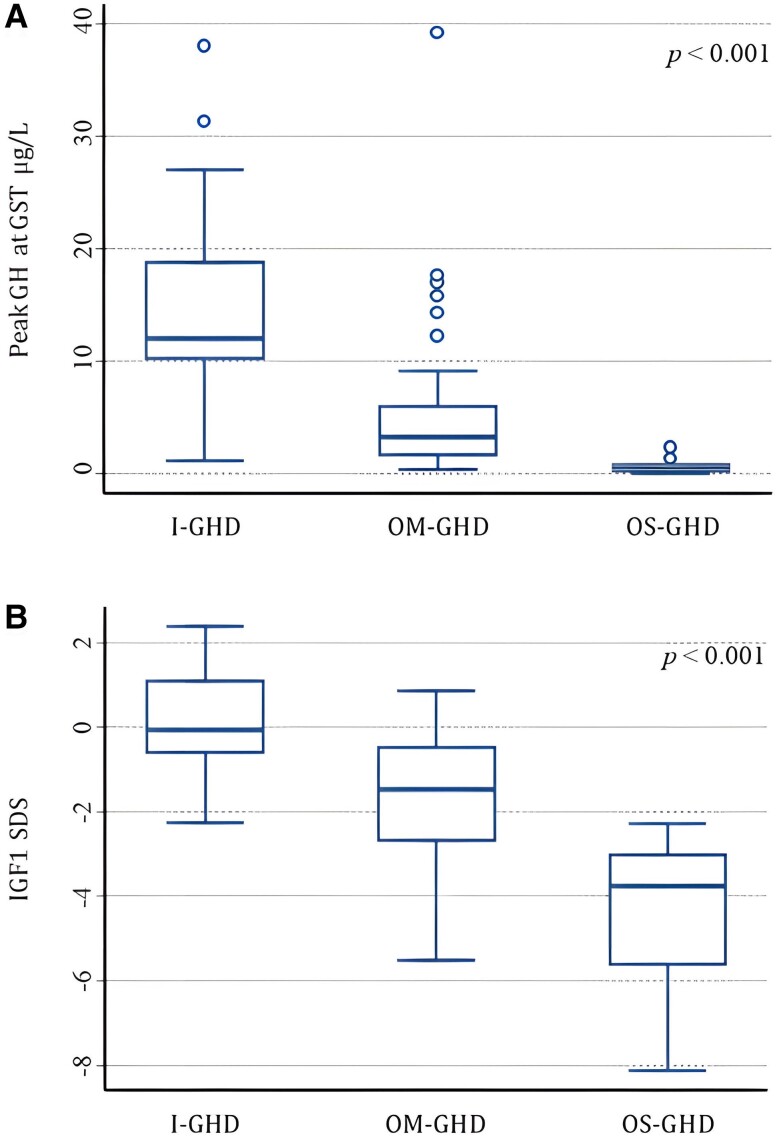
(A) Box plots show median (thick line), the IQR, delimited by the first and third quartile (margins of the box), and the range of values between ± 1.5 IQR from the margin of the box (whiskers) of peak GH at GST in the 3 groups: I-GHD, OM-GHD, OS-GHD. *P*-value from the Kruskal–Wallis nonparametric test. Outliers are also shown for each group. (B) Box plots show median (thick line), first and third quartile (margins of the box), minimum and maximum values of IGF-1 SD score at retesting in the 3 groups: I-GHD, OM-GHD, OS-GHD. *P*-value from the Kruskal–Wallis nonparametric test is indicated. Abbreviations: GHD, GH deficiency; GST, glucagon stimulation test; I-GHD, idiopathic growth hormone deficiency; IQR, interquartile range; OM-GHD, organic moderate GHD; OS-GHD, organic severe GHD.

The median GH peak to ITT was 6.31 μg/L (IQR 1.73; 15.05) and to GST 6.85 μg/L (IQR 1.73; 12.41). A GH value < 6 μg/L to ITT was found in 46 patients: in 17 of 18 OS-GHD (94.5%), 25 of 35 OM-GHD (71.5%), and 4 of 44 I-GHD (9%) patients. GH peak to GST was lower in OS-GHD and OM-GHD groups compared to I-GHD (0.71, IQR 0.27;1.73 vs 3.24, IQR 1.67;5.94 vs 12.03, IQR 10.32;18.77, respectively, *P* = .0001) (Fig. 2A). Likewise, IGF-1 SDS was lower in the OS-GHD and OM-GHD groups compared to I-GHD (−2.28, IQR −3.77; −1.31 vs −1.96, IQR −2.83; −0.585 vs 0.00, IQR −0.59;0.91, respectively, *P* = .0001) (Fig. 2B). In the OS-GHD group, 11 patients (61%) had serum IGF-1 levels < −2 SDS, and 6 patients (33%) had serum IGF-1 between −2 and 0 SDS. In 2 patient in the OS-GHD group, IGF-1 SDS value was missing. In the OM-GHD group, 17 patients (48.6%) had serum IGF-1 levels < −2 SDS, 14 patients (40%) were between −2 and 0 SDS, and 4 patients (11.4%) had values of IGF-1 > 0 SDS. Thirteen CCS patients in the OM-GHD group had IGF-1 levels < −2 SDS (48.1%), 11 patients had IGF-1 levels between −2 and 0 SDS (40.7%), and 3 patients had IGF-1 levels ≥0 SDS (11.1%). Ten of 14 CCS patients in the OS-GHD group had IGF-1 levels < −2 SDS (71.4%) and 4 patients had IGF-1 levels between −2 and 0 SDS (28.6%).

Based on the GH response to ITT < or ≥6 μg/L at retesting, 51 patients (52.6% of the entire cohort) showed a GH peak ≥6 μg/L; 2% (n = 1) belonged to the OS-GHD group, 19.6% (n = 10) to the OM-GHD group, and 78.4% (n = 40) to the I-GHD group. Six of 10 patients in the OM-GHD group with a GH peak ≥6 μg/L to ITT were CCS.

The biochemical data showed a significantly lower blood glucose in OS-GHD patients and in those of OM-GHD group compared to I-GHD patients (*P* = .0002), as indicated in Table 2. Insulin values were within the normal range in all groups and not statistically different.

### Biochemical **C**haracteristics **D**uring the **G**lucagon **T**est

**Figure 3.**
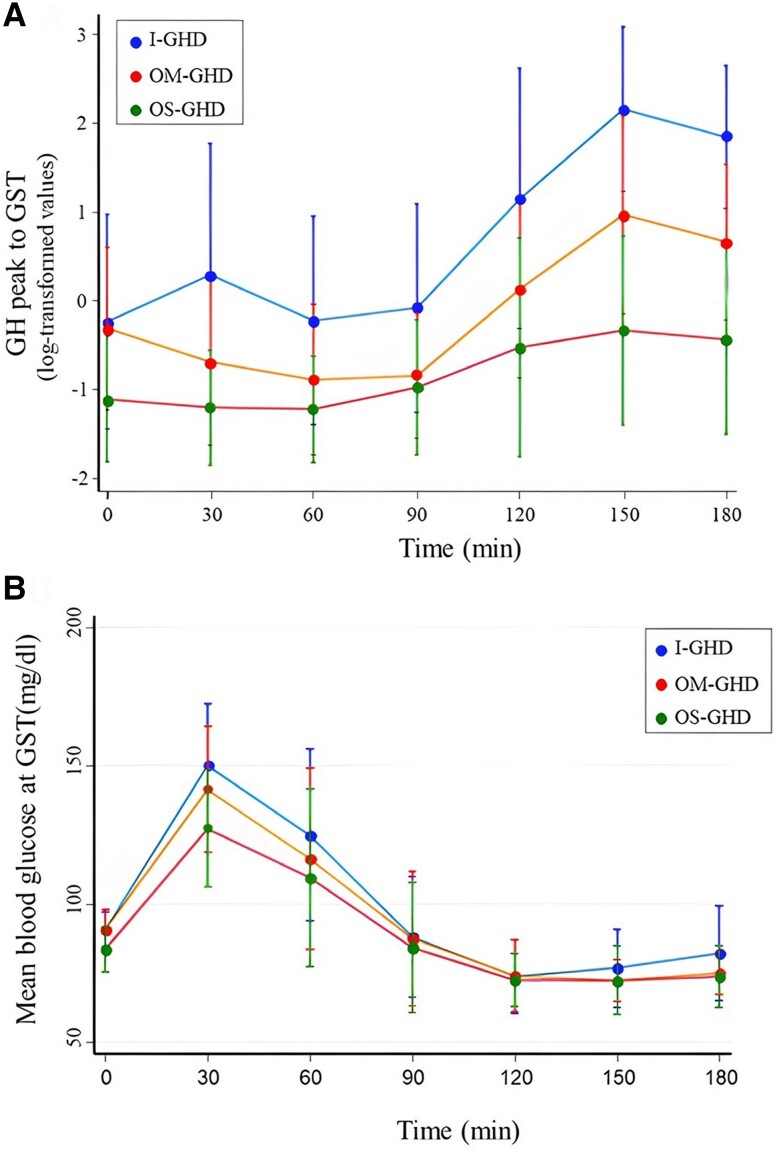
(A) Mean (SD) GH values curve at each time point of GST in patients withI-GHD, OM-GHD, OS-GHD. (B) Mean (SD) blood glucose value curve at each time point of GST in patients with I-GHD, OM-GHD, OS-GHD. Abbreviations: GHD, GH deficiency; GST, glucagon stimulation test; I-GHD, idiopathic growth hormone deficiency; OM-GHD, organic moderate GHD; OS-GHD, organic severe GHD.

The highest mean peak GH value (7.12 ± 7.26 μg/L) to GST was recorded 150 minutes after glucagon injection either in the entire population and in all groups (I-GHD, OM-GHD, OS-GHD). Specifically, the I-GHD group showed the highest mean peak GH value (11.61 ± 7.55 μg/L), while the OS-GHD group showed the lowest mean (1.27 ± 2.31 μg/L) (Fig. 3A). Blood glucose reached its highest mean value between 30 and 60 minutes after glucagon injection in the entire cohort and in each of the 3 groups, with a mean peak value of 150 mg/dL in I-GHD after 30 minutes (Fig. 3B), while the lowest mean level was recorded at 150 minutes in the OM-GHD and OS-GHD groups (72 mg/dL). Baseline blood glucose was significantly lower in the OS-GHD group (mean value 83.7 ± 8.2 mg/dL) compared to the other groups (mean values: 90.8 ± 7.2 mg/dL in OM-GHD and 90.8 ± 6.4 mg/dL in I-GHD patients, *P* = .001) and after 30 minutes (mean value 127.3 ± 21.3 vs 141.4 ± 22.7 mg/dL in OM-GHD and 150.3 ± 22.3 mg/dL in I-GHD patients, *P* = .002); the I-GHD group had the most rapid increase in blood glucose.

### A**ssociation Between GH Peak to ITT, GH Peak to GST and Biochemical Variables**

A strong positive correlation was observed between ITT and GST for GH peak values in the entire cohort (ρ = 0.85, *P* < .001) and in each group, with stronger correlation in OS-GHD (ρ = 0.95, *P* < .001).

**Figure 4.**
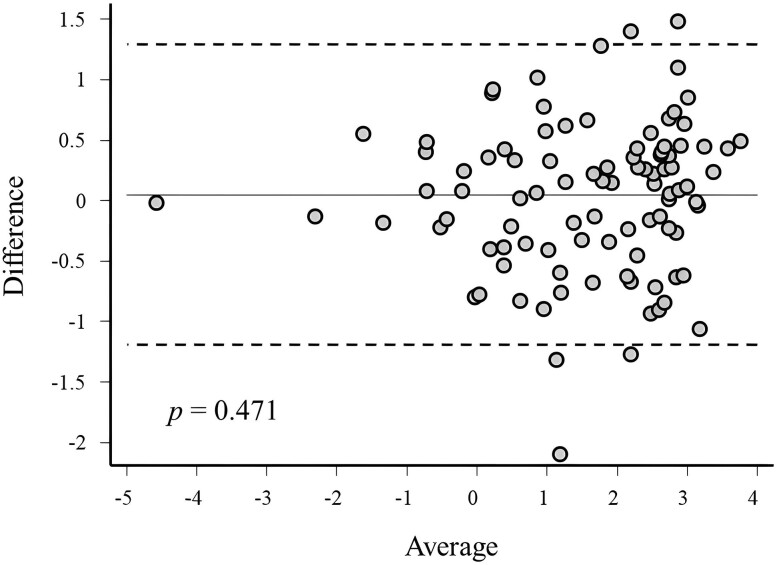
Agreement between peak GH values at the ITT and at the GST, evaluated by the Bland and Altman plot. The *P*-value associated with the Pitman's test is shown. Abbreviations: GST, glucagon stimulation test; ITT, insulin tolerance test.

Bland and Altman analysis showed a high consistency of results from the GH peak measures after ITT and GST (Fig. 4). Accordingly, a very high agreement was also estimated by the ICC analysis (92.4%, 95% CI 88.8-94.8). There was no correlation between GH peak to GST or ITT and the postirradiation time interval (GST, ρ = 0.23, *P* = .211; ITT, ρ = 0.25, *P* = .174).

GH peak to ITT showed a significant positive correlation with IGF-1 SDS in the entire cohort (ρ = 0.70, *P* < .001), in OS-GHD (ρ = 0.89, *P* < .001), and in OM-GHD (ρ = 0.54, *P* < .001) groups, even after excluding female patients who were receiving HRT (data not shown).

GH peak to GST significantly correlated with serum IGF-1 SDS levels in the entire cohort (ρ = 0.68, *P* < .001) even after excluding female patients who were receiving HRT, in OS-GHD (ρ = 0.81, *P* < .001), and in OM-GHD (ρ = 0.50, *P* = .002) groups.

GH peak to GST correlated with serum blood glucose (r = 0.38, *P* < .001) in the entire cohort. A significant inverse correlation between GH peak to GST and BMI SDS was found only in the entire cohort (ρ = −0.36, *P* < .001) but not in the analysis stratified by GHD groups.

### ROC Analyses for GH **P**eak **A**fter GST **T**esting and IGF-I

**Figure 5.**
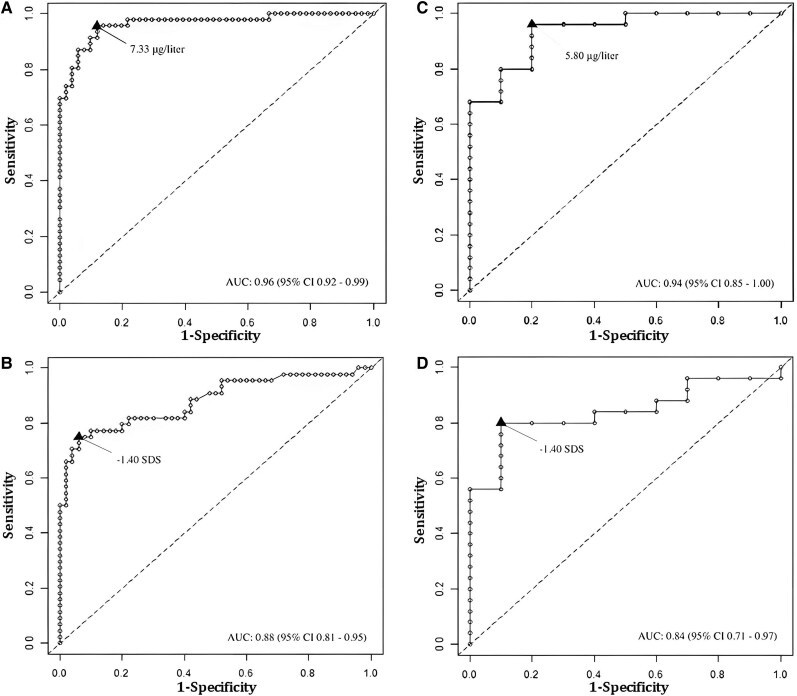
(A-C) ROC curve analyses of peak GH to GST in the entire cohort (A) and in OM-GHD group (C). (B-D) ROC curve analyses of IGF-1 SDS in the entire cohort (B) and in OM-GHD group (D). Abbreviations: OM-GHD, organic moderate GHD; ROC, receiver operating curve; SDS, SD score.

The results of ROC curve analysis of GST (Fig. 5A) showed a GH peak value of 7.3 μg/L as the optimal cutoff (95% CI 4.15-8.91; sensitivity 95.7%, specificity 88.2%, PPV = 88.0%, NPV = 95.7%), able to correctly classify 91.8% of the entire cohort; the AUC was 0.94 (95% CI 0.93-1.00). The optimal cutoff value for IGF-1 was −1.4 SDS (95% CI −1.94 to 0.77; sensitivity 75%, specificity 94%, PPV = 91.7%, NPV = 81.0%) (Fig. 5B) that correctly classified 85.1% of the study population; the AUC was 0.88 (95% CI 0.81–0.95).

Subgroup analyses were possible only in OM-GHD patients (highly likelihood GHD), due to the insufficient sample size in the OS-GHD group. The proportion of correctly classified patients at the optimal threshold (91.4%) was obtained for a GH peak cutoff of 5.8 μg/L (95% CI 3.16-7.39; sensitivity, 96.0%, specificity 80.0%, PPV 92.3%, NPV = 88.9%) (Fig. 5C). Applying such a cutoff to the entire cohort, results were very similar to those observed at the optimal cutoff, namely sensitivity 91.3%, specificity 90.2%, PPV = 89.4%, and NPV = 92.0%).

In this group, the same IGF-1 cutoff value of −1.4 SDS was found (95% CI −2.47 to −0.72; sensitivity 80%, specificity 90%, PPV 95.2%, NPV = 64.3%) (Fig. 5D), which correctly classified 82.9% of the patients with an AUC of 0.84 (95% CI 0.71–0.97).

### Association **B**etween GH **P**eak to ITT, GH **P**eak to GST, and **P**atients**’ C**haracteristics at **T**ransition

Associations between GH defect, assessed by the peak to ITT, and the main patient's characteristics at the transition (namely, peak GH to GST, IGF-1 SDS, BMI SDS, and brain MRI result) is reported in Table 3. Higher values of both peak GH to GST and IGF-1 SDS were associated with a lower probability of GH deficit, whereas a positive association was observed with the BMI SDS. MRI results were strongly associated with GHD in univariable analysis, but this association was reduced and no longer significant in multivariable analysis. The association between GHD, evaluated by the peak to GST at 2 different cutoffs (7.3 and 5.8 μg/L) and main patients’ characteristics at transition, is shown in Table 4. At both cutoffs, BMI SDS was not associated with GH peak in multivariable regression analysis. Furthermore, regression analysis stratified by patient groups (I-GHD, OM-GHD, OS-GHD) revealed no statistically significant association; the limited sample size, particularly among patients with OS-GHD, prevented reliable conclusions from being drawn. In particular, ROC analysis based on BMI levels (≤1, 1-2, ≥2 SDS) has a remarkably high level of accuracy among patients with a normal BMI (≤1 SDS, n = 61) with an AUC of 0.99 (95% CI 0.98-1.0); the optimal cutoff in these patients was 5.07 μg/L (95% CI 3.7-6.7, sensitivity 95%, specificity 97%). In patients with overweight (1-2 SDS, n = 21), the AUC was 0.93 (95% CI 0.82-1.0), with an optimal cutoff of 7.3 μg/L (95% CI 2.2-9.5, sensitivity 89%, specificity 83%). Due to permanent GHD in all 15 patients with obesity (BMI ≥ 2 SDS), ROC analysis could not be conducted.

**Table 3.** Logistic regression model to assess the association between GH deficit and patient characteristics at the transition

	Univariable model			Multivariable model		
Patient characteristics	exp (β)	95% CI	*P*	exp (β)	95% CI	*P*
Peak GH to GST	0.566	0.455-0.706	<.001	0.635	0.490-0.824	<.001
BMI SDS	2.067	1.402-3.047	<.001	2.747	1.093-6.901	.009
IGF-1 SDS	0.277	0.163-0.471	<.001	0.366	0.160-0.840	.004
MRI	38.18	11.23-129.8	<.001	3.128	0.411-23.78	.267

Analysis by group was not feasible due to very sparse data.

Abbreviations: BMI, body mass index; CI, confidence interval; exp(β), exponentiated regression coefficient (corresponding to the odds ratio for categorical variables); GST, glucagon stimulation test; MRI, brain magnetic resonance imaging (normal vs abnormal result); SDS, SD score.

**Table 4.** Logistic regression model to assess the association between GH deficit, assessed by GH GST and patient characteristics at the transition

	Univariable model			Multivariable model		
Patient characteristics	exp (β)	95% CI	*P*	exp (β)	95% CI	*P*
Cut-off: 7.34						
BMI SDS	1.743	1.228-2.472	<.001	1.427	0.843-2.414	.175
IGF-1 SDS	0.335	0.210-0.532	<.001	0.504	0.298-0.851	.003
MRI	30.96	10.09-94.948	<.001	12.61	3.455-46.02	<.001
Cut-off: 5.8						
BMI SDS	1.595	1.143-2.226	.003	1.248	0.737-2.112	.405
IGF-1 SDS	0.318	0.197-0.514	<.001	0.476	0.277-0.817	.001
MRI	43.00	12.48-148.1	<.001	13.59	3.526-52.36	<.001

Analysis by group was not feasible due to very sparse data.

Abbreviations: BMI, body mass index; CI, confidence interval; exp(β), exponentiated regression coefficient (corresponding to the odds ratio for categorical variables); GST, glucagon stimulation test; MRI, brain magnetic resonance imaging (normal vs abnormal result); SDS, SD score.

### Discordance Between ITT and GST Response

Forty-three of the 46 patients (93.4%) with GH peak to ITT < 6 μg/L had peak GH at GST < 7.3 μg/L and 42 patients had GH peak at GST < 5.8 μg/L; 4 patients showed discordant responses with a peak GH at ITT < 6 μg/L but above 5.8 μg/L at GST, as shown in Table 5.

**Table 5.** Patients with discordance in GH response between ITT and GST

No.	Group	Sex	MRI result	Diagnosis	Radiotherapy	No. of pituitary defects*^a^*	Height SDS	BMI SDS	Tanner stage	Years at retesting	Peak GH at ITT μg/L	Peak GH at GST μg/L	IGF-1 SDS
Patients with peak GH < 5 μg/L at ITT and peak GH ≥ 5.8 μg/L at GST													
1	OM-GHD	M	N	ALL	TBI	2	−2	1.1	4	15.9	1.1	8.72	−2.31
2	I-GHD	M	N			1	−1.4	1.5	5	18.9	5.34	7.32	
3	I-GHD	F	N			1	−1.4	2.8	5	15.4	4.57	15.9	1.99
4	I-GHD	F	N			1	−0.9	2.9	5	18.0	3.56	6.85	−0.32

Abbreviations: ALL, acute lymphoblastic leukemia; BMI, body mass index; GHD, GH deficiency; GST, glucagon stimulation test; I-GHD, idiopathic GHD; ITT, insulin tolerance test; MRI, magnetic resonance imaging; N, normal hypothalamic pituitary anatomy; OM-GHD, organic moderate GHD; TBI, total body irradiation; SDS, SD score.

^*a*^GHD and another defect.

### Diagnostic Role of IGF-I SDS and GH Testing Based on the AACE Recommendations

Patients with permanent GHD classified based on IGF-1 SDS levels of <−2SDS, ≥−2 and <0 SDS, and ≥0 SDS and GH peaks after ITT (as a gold standard) and the 2 different GST cutoffs obtained from the ROC analysis are shown in Table 6. It is worth highlighting that 5 patients with permanent GHD in the OS-GHD group have IGF-1 values ≥−2 SDS, despite the presence of 3 pituitary hormonal defects due to organic disease or congenital defects.

**Table 6.** IGF-1 SDS values in patients with permanent GHD of different etiologies based on ITT and GST cutoffs

	IGF-1 < −2 SDS	IGF ≥ −2 and <0 SDS	IGF-1 ≥ 0 SDS
Peak GH ITT < 6 μg/L (n) OS-GHD (n = 16)*^a^*	11	5	0
OM-GHD (n = 25)	15	9	1
I-GHD (n = 3)*^a^*	1	1	1
Total (n = 44)	27	15	2
Peak GH GST < 7.3 μg/L (n)			
OS-GHD (n = 16)*^a^*	11	5	0
OM-GHD (n = 27)	14	11	2
I-GHD (n = 5)	1	2	2
Total (n = 48)	26	18	4
Peak GH GST < 5.8 μg/L (n)			
OS-GHD (n = 16)*^a^*	11	5	0
OM-GHD (n = 26)	14	10	2
I-GHD (n = 4)	1	1	2
Total (n = 46)	26	16	4

Abbreviations: GHD, GH deficiency; GST, glucagon stimulation test; I-GHD, idiopathic GHD; ITT, insulin tolerance test; OM-GHD, organic moderate GHD; OS-GHD, organic severe GHD; SDS, SD score.

^*a*^Missing: n = 1 in the OS-GHD group; n = 1 in the I-GHD group.

Patients with permanent GHD were also classified according to AACE cutoff for GST based on BMI range for healthy weight (<25 kg/m^2^), overweight (≥25 kg/m^2^ and <30 kg/m^2^), and obesity (≥30 kg/m^2^) (Table 7) and IGF-1 SDS levels. Peak GH cutoffs of 3 and 1 μg/L were used in the OM-GHD and I-GHD groups, respectively, for overweight patients based on their pretest probability of GHD.

**Table 7.** Distribution of patients based on GH cutoffs and IGF-I SDS and BMI proposed by the American Association of Clinical Endocrinologists

		BMI < 25 kg/m^2^			BMI ≥ 25 and <30 kg/m^2^*^a^*			BMI ≥ 30 kg/m^2^		
		IGF-1 < −2 SDS (n)	IGF-1 ≥ −2 and <0 SDS (n)	IGF-1 ≥ 0 SDS (n)	IGF-1 < −2 SDS (n)	IGF-1 ≥ −2 and <0 SDS (n)	IGF-1 ≥ 0 SDS (n)	IGF-1 < −2 SDS (n)	IGF-1 ≥ −2 and <0 SDS (n)	IGF-1 ≥ 0 SDS (n)
Cut-off GH μg/L										
OS-GHD	ITT < 5	5	1		4	2		2	1	
	GST ≤3	5	1		4	2				
	GST ≤1							2	0	
OM-GHD	ITT < 5	13	6	0	2	2	1		1	
	GST ≤3	8	4	0	2	0	0			
	GST ≤1								0	
I-GHD	ITT < 5	1	0	0		0			1	1
	GST ≤3	1	0	0						
	GST ≤1					0			0	0

Abbreviations: BMI, body mass index; GHD, GH deficiency; GST, glucagon stimulation test; I-GHD, idiopathic GHD; ITT, insulin tolerance test; OM-GHD, organic moderate GHD; OS-GHD, organic severe GHD; SDS, SD score.

^*a*^n = 1 missing in OS-GHD group.

## Discussion

The validation for the GST in GHD patients transitioning from pediatric to adult care is still lacking, and the search for an accurate and safe alternative test to ITT has been emphasized in the need to reevaluate the function of the GH/IGF-I axis in subjects with CO-GHD at risk of permanent GHD (18, 22, 23, 34, 45). While the ITT represents a gold standard in the diagnosis of GHD after adult height achievement based on the cutoffs of 5 or 6 μg/L adopted by the international societies (18, 34, 45, 46), the diagnostic accuracy of other tests including GHRH plus arginine or GHRH alone or arginine remains questionable (22-24). Of note, data on provocative tests with macimorelin and glucagon, although proposed by the AACE, have not yet been reported during transition (34). The GST has been reported as a reliable alternative for the diagnosis of GHD in adults and in the elderly and could be a promising option also in subjects with CO-GHD after adult height achievement (47-55).

Several studies in adults demonstrated that GH cutoff values of 2.5 (54) or 3 μg/L (48, 49) after GST provided optimal sensitivity and specificity regardless of age and sex (48) or hypothalamic origin of GHD (47). Using ROC analysis, a lower GH cutoff value near 1.0 μg/L after GST provided optimal (52) or acceptable (56) sensitivity and specificity. Interestingly, patients with hypothalamic-pituitary disease and 1 to 2 or ≥3 pituitary defects showed an optimal GH cutoff value of 1.0 (92% sensitivity, 100% specificity) or 2.0 μg/L (96% sensitivity, 100% specificity) depending on the administration of a fixed (1 or 1.5 mg >90 kg body weight) or weight-adjusted glucagon injected dose (0.03 mg/kg, maximum 3 mg), respectively (57). Conversely, a “falsely normal” peak GH response (between 8 and 16 μg/L) has been reported in children with congenital hypopituitarism and pituitary stalk interruption syndrome (58), suggesting its potency compared to other provocative tests. This finding also raises the question of whether a distinct cutoff is needed to diagnose GHD during transition.

In this study, we evaluated the role of GST as an alternative to ITT for the diagnosis of permanent GHD in 97 patients with CO-GHD of different etiologies after adult height achievement and found a robust positive correlation between GH peaks to GST and ITT. A strong concordance between the 2 measures, as demonstrated by an ICC around 92% and corresponding to what was reported in 265 patients with severe GH deficiency patients (GH response <3) in the KIGS database (59).

A cutoff value of 7.3 μg/L obtained by ROC curve analysis was appropriate for the diagnosis of GHD during transition independently of the hypothalamic-pituitary origin of the GH defect. In addition, we found that the best cutoff value for GHD diagnosis in the most challenging group of patients with high pretest probability of permanent GHD/ OM-GHD was 5.8 μg/L. Indeed, using this cutoff the diagnostic accuracy was reached in 92.3% of patients with OM-GHD, with a sensitivity of 96.0% and specificity of 80.0%. In addition, by applying such a cutoff to the entire cohort, the accuracy did not differ (89.4%) with a sensitivity of 91.3%, specificity of 90.2%, and AUC of 0.94 suggesting that the cutoff of 5.8 μg/L is reliable in the diagnosis of GHD patients at high risk of permanent GHD, independently of the hypothalamic-pituitary etiology (ie, brain tumor, irradiation, congenital abnormalities).

The diagnosis of GHD was confirmed after ITT in 47.4% of our cohort, in 94.4% of the OS-GHD group, in 71.4% of the OM-GHD group, and in 9% of the I-GHD group. Similarly, 48.5% of our cohort showed a GH peak at the GST of less than 5.8 μg/L, 94.4% of the OS-GHD group (82.3% of which had brain tumors), 74.3% of the OM-GHD group (84.6% of which were CCS or underwent CSI or TBI), and 9% of the I-GHD group. GH secretion testing is known to be challenging in CCS, especially when neurological outcomes recommend against ITT and brain irradiation against GHRH plus arginine testing (22). It is worth pointing out that GHD was reconfirmed in 85.4% of CCS with ITT and in 87.8% with GST by using the cutoff point of 5.8 μg/L, reaching a concordance of 82.9% between the 2 tests. The response to GST was independent of the postirradiation time interval reported for GHRH plus arginine (23, 25-27). Overall, the results obtained by ITT and GST suggest that OS-GHD, OM-GHD, CCS, or MPHD subjects are those in whom GHD is most likely to be confirmed during transition, as observed in previous studies (49, 60).

The GH cutoffs after GST in our cohort appeared accurate after stratifying our patients based on BMI. Obesity was identified in approximately one-third of the OS-GHD group and in 20% of the OM-GHD group, whereas it was absent in the 51 patients with normal peak GH response to ITT. Previous studies in adults have reported discordant correlations between GH peak values after GST and obesity, raising the question of the need of considering BMI in the interpretation of GH peak results (50-57, 59). By excluding patients with ≥3 hypothalamic-pituitary defects, Diri et al (56) found a significant negative correlation between BMI and peak GH at GST, while in a cohort of more than 500 adults, Yuen et al (50) confirmed a negative correlation with peak GH, similar to the one we found in the cohort overall (r = 0.36) but not in our groups individually. The paper by Toogood et al (59) also confirmed a negative yet complex curvilinear relation between BMI and GH peak in more than 650 adults, leading, however, to an overall small final effect of about 0.25 µg/L. In contrast, others found no correlation (54, 57), and some only observed it in healthy controls (49, 52).

In our study, the BMI SDS was predictive of GH response to GST in univariate regression analyses but not in multivariable models where the abnormal MRI features and IGF-1 SDS levels remained the only significant and independent determinants. Indeed, in the multivariate analysis, the correlation between BMI SDS and GH levels appears to be mainly driven by the significant correlation between BMI SDS and the severity of GHD, where the OS-GHD group shows the highest values, while the lowest values were observed in I-GHD patients. Although the association was no longer significant after stratification by patient or group, the small sample size among patients with OS-GHD (n = 18) prevented drawing definitive conclusions.

We found that IGF-1 SDS shows a positive correlation with peak GH at ITT and GST both in the entire cohort and in subgroups, and the analysis of ROC curves demonstrated the best diagnostic accuracy for a cutoff IGF-1 of −1.4 SDS in both the entire cohort and in the OM-GHD group, with sensitivity and specificity ranging from 75% to 80% and 90% to 94%, respectively. When the analyses were conducted after excluding patients taking oral estrogens, there was a minimal, nonsignificant change in sensitivity at the optimal cutoff (sensitivity 74%). However, it is important to highlight that only 2 patients received oral estrogens, both in the OS-GHD group, suggesting that the potential impact of this confounding factor cannot be assessed in the study population. The lower sensitivity of IGF-1 SDS compared to GH cutoffs is also confirmed by finding levels lower than −2 SDS in only 45.7% of OM-GHD patients and 61.1% of OS-GHD patients. Interestingly, in the CCS cohort, only 41.5% had IGF-1 < −2 SDS and 7.3% ≥ 0 SDS. The optimal cutoffs at GST, as determined by ROC analysis, were equally effective in identifying permanent GHD in all groups, regardless of IGF-1 SDS values, except for 1 patient with OM-GHD. The latter had a high pretest probability of permanent GHD secondary to acute lymphoblastic leukemia and TBI, central hypothyroidism, and peak GH of 1.1 μg/L at ITT and 8.72 μg/L at GST, with an IGF-I value of −2.32 SDS (Table 5).

The 2019 AACE guidelines (34) recommend using a GH cut-point of 3 µg/L for normal-weight patients (BMI <25 kg/m^2^), a GH cut-point of 1 µg/L in patients with obesity (BMI >30 kg/m^2^), and a GH cut-point of 3 or 1 µg/L in overweight patients (BMI 25-30 kg/m^2^) based on the high or low pretest probability. Applying the AACE-recommended GH peak cutoffs to the entire cohort led the GST to (1) accurately identify 93.3% of the OS-GHD group (which refers to congenital, genetic, or organic disease with ≥ 3 pituitary defects), regardless of the presence of normal weight or overweight; 2) identify only 56% of OM-GHD patients (refers to organic disease or congenital anomalies with 0, 1, or 2 pituitary defects) with permanent GHD that had been diagnosed by ITT (the diagnosis of GHD would be missed in 11 patients regardless of BMI SDS and IGF-1 SDS); 3) identify one-third of the patients diagnosed as permanent I-GHD by ITT. According to the international recommendations, we confirmed that both patients with organic OS-GHD who have 3 or more defects and those with idiopathic GHD and IGF-1 ≥ 0 SDS due to the low pretest probability of permanent GHD do not require retesting.

A limitation of the current study is represented by the insufficient sample size in patients with OS-GHD, where indeed retesting is not recommended. Another potential limitation is that our protocol establishes a final sampling time of 180 minutes after glucagon administration; some patients may show the highest level of GH secretion between 210 and 240 minutes later (52, 56, 61).

It is important to note that heterogeneity still exists in currently used tests, which may prevent the relevancy of the proposed cutoff, although this limitation applies to all recommended cutoffs for the diagnosis of GHD. Furthermore, it is essential to highlight that, in order to minimize the variation between assay methods, the AACE recommends (34) that all GH assays be calibrated against the international reference standard 98/574 (National Institute for Biological Standards and Control). Another important aspect to consider is the value of the suggested GST cutoff in patients with impaired glucose tolerance and diabetes mellitus, conditions that were absent in our cohort. According to the findings of Wilson et al (55), the absence of hypoglycemia during GST stimulus could be a contributing factor to the impaired GH response to GST.

When considering safety and reproducibility, the benefits of using GST outweigh the disadvantages, which include the long duration of the test with multiple blood draws and the requirement of an intramuscular injection. Nausea and/or vomiting described in adults were never reported in our cohort. A total of 10 patients were found to have blood glucose levels below 54 mg/dL during the test. This accounted for 6.8% of the I-GHD group, 11.4% of the OM-GHD group, and 16.6% of the OS-GHD group. However, none of these patients experienced symptomatic hypoglycemia.

**Figure 6.**
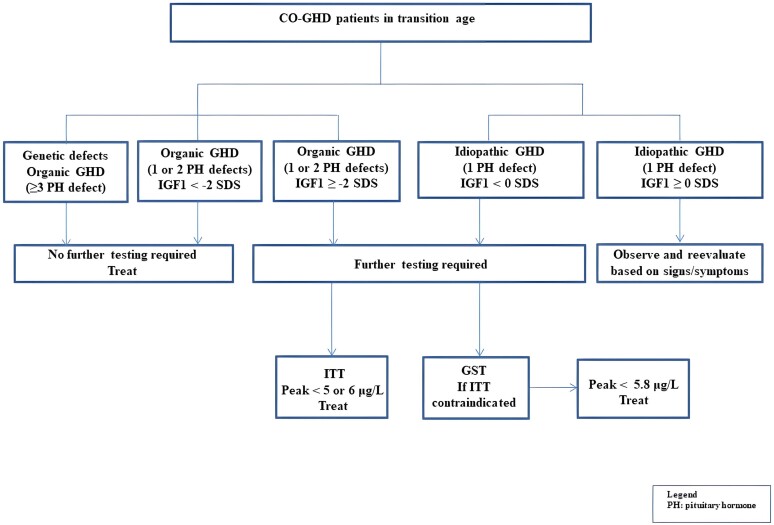
Algorithm for the evaluation of GH function in childhood-onset GHD after adult at the time of transition. Abbreviation: GHD, GH deficiency.

To our knowledge, this is the first study to establish threshold values for GST in the diagnosis of GHD in the transition age. Our data confirm that GST is a safe alternative to ITT for assessing GH secretion in young adults with CO-GHD after attainment of adult height and that patients who demonstrated peak GH < 5.8 μg/L can restart rhGH therapy. Based on our results, we propose to retest with GST all CO-GHD patients with organic GHD (brain tumors, congenital anomalies, irradiation to the hypothalamic/pituitary region) with 0, 1, or 2 pituitary hormone defects with IGF-1≥ −2 SDS, and patients with idiopathic GHD with IGF-1 < 0 SDS (Fig. 6).

## Data Availability

The data sets produced through the current study are not publicly available but are available from the corresponding author on reasonable request.
